# POLG2 deficiency causes adult‐onset syndromic sensory neuropathy, ataxia and parkinsonism

**DOI:** 10.1002/acn3.361

**Published:** 2016-11-16

**Authors:** Lionel Van Maldergem, Arnaud Besse, Boel De Paepe, Emma L. Blakely, Vivek Appadurai, Margaret M. Humble, Juliette Piard, Kate Craig, Langping He, Pierre Hella, François‐Guillaume Debray, Jean‐Jacques Martin, Marion Gaussen, Patrice Laloux, Giovanni Stevanin, Rudy Van Coster, Robert W. Taylor, William C. Copeland, Eric Mormont, Penelope E. Bonnen

**Affiliations:** ^1^ Centre de génétique humaineUniversité de Franche‐ComtéBesançonFrance; ^2^ Metabolic UnitCentre of Human GeneticsUniversity HospitalLiègeBelgium; ^3^ Department of Molecular and Human GeneticsBaylor College of MedicineHoustonTexas; ^4^ Department of PediatricsDivision of Child Neurology & MetabolismGhent University HospitalBelgium; ^5^ Wellcome Trust Centre for Mitochondrial ResearchInstitute of NeuroscienceNewcastle UniversityNewcastle upon TyneUnited Kingdom; ^6^ Mitochondrial DNA Replication GroupNational Institute of Environmental Health SciencesDurhamNorth Carolina; ^7^ Department of NeurologySambre and Meuse Regional HospitalNamurBelgium; ^8^ Born‐Bunge FoundationUniversity of AntwerpBelgium; ^9^ Inserm U1127CNRS UMR7225Sorbonne UniversitésUPMCParisFrance; ^10^ Institut du Cerveau et de la Moelle épinièreHopital Pitié‐SalpêtrièreParisFrance; ^11^ Ecole Pratique des Hautes EtudesPSL UniversitéLaboratoire de neurogénétiqueF‐75013ParisFrance; ^12^ Université catholique de LouvainCHU UCL NamurDepartment of NeurologyB5530YvoirBelgium; ^13^ UCL Institute of Neuroscience (IoNS)B1200BrusselsBelgium

## Abstract

**Objective:**

Mitochondrial dysfunction plays a key role in the pathophysiology of neurodegenerative disorders such as ataxia and Parkinson's disease. We describe an extended Belgian pedigree where seven individuals presented with adult‐onset cerebellar ataxia, axonal peripheral ataxic neuropathy, and tremor, in variable combination with parkinsonism, seizures, cognitive decline, and ophthalmoplegia. We sought to identify the underlying molecular etiology and characterize the mitochondrial pathophysiology of this neurological syndrome.

**Methods:**

Clinical, neurophysiological, and neuroradiological evaluations were conducted. Patient muscle and cultured fibroblasts underwent extensive analyses to assess mitochondrial function. Genetic studies including genome‐wide sequencing were conducted.

**Results:**

Hallmarks of mitochondrial dysfunction were present in patients’ tissues including ultrastructural anomalies of mitochondria, mosaic cytochrome *c* oxidase deficiency, and multiple mtDNA deletions. We identified a splice acceptor variant in *POLG2,* c.970‐1G>C, segregating with disease in this family and associated with a concomitant decrease in levels of POLG2 protein in patient cells.

**Interpretation:**

This work extends the clinical spectrum of POLG2 deficiency to include an overwhelming, adult‐onset neurological syndrome that includes cerebellar syndrome, peripheral neuropathy, tremor, and parkinsonism. We therefore suggest to include *POLG2* sequencing in the evaluation of ataxia and sensory neuropathy in adults, especially when it is accompanied by tremor or parkinsonism with white matter disease. The demonstration that deletions of mtDNA resulting from autosomal‐dominant *POLG2* variant lead to a monogenic neurodegenerative multicomponent syndrome provides further evidence for a major role of mitochondrial dysfunction in the pathomechanism of nonsyndromic forms of the component neurodegenerative disorders.

## Introduction

Mitochondrial dysfunction caused by disturbances of mtDNA integrity gives rise to a diverse array of neurological, neuromuscular, and multisystem disorders. The clonal expansion of multiple mtDNA deletions is typically associated with late‐onset primary mitochondrial disease characterized by autosomal‐dominant progressive external ophthalmoplegia (adPEO) as the predominant clinical feature.^1^ MtDNA is replicated by the DNA polymerase gamma complex, which is composed of a monomeric catalytic subunit encoded by *POLG* and a dimeric accessory subunit encoded by *POLG2*. Pathogenic *POLG* variants are a common cause of primary mitochondrial disorders typically resulting in adPEO, but can also lead to a phenotypic spectrum manifesting variably across brain, muscle, and/or liver (OMIM 157640, 258450, 203700, 613662, and 607459).

Few families have been reported to have disease due to variants in *POLG2*. The first reported case of *POLG2* disease was documented in a patient with adult‐onset autosomal dominant PEO in whom multiple mtDNA deletions were detected.^2^ The second identified case of *POLG2* disease was a patient who developed ptosis at age 30, but not ophthalmoplegia, and proximal and distal myopathy in her late forties. Muscle biopsy showed clonally expanded multiple mtDNA deletions.^3^ This family included a brother who possessed the same *POLG2* variant and showed no signs of disease at age 57, indicating possible incomplete penetrance. Subsequently, three cases with pediatric‐onset autosomal dominant disease with varying phenotypes have been reported: a young adult with disease onset of adPEO in late teens and two unrelated infants presenting with hypotonia, seizures, and liver disease.^4^ With only a small number of cases reported, it is clearly evident that *POLG2*‐related disorders display a spectrum of clinical phenotypes with a wide range of age of onset.

We describe a severe neurological syndrome in a large family with cerebellar ataxia, axonal sensory ataxic neuropathy, and tremor. Some subjects also presented with parkinsonism, seizures, cognitive decline, and ophthalmoplegia. Most died within 15 years of onset of clinical symptoms of disease. Genetic studies revealed a *POLG2* splice acceptor variant segregating with disease, leading to loss of *POLG2* protein in patient cells. This work extends the clinical spectrum observed with *POLG2* disease and lends further support for the role of the accumulation of mtDNA deletions in neurodegeneration.

## Materials and Methods

### Muscle histopathology and mtDNA analyses

Cryostat sections (10 *μ*m) were cut from transversely orientated muscle blocks and subjected to standard histological and histochemical analysis including COX, succinate dehydrogenase (SDH), and sequential COX‐SDH oxidative enzyme reactions. Large‐scale mtDNA rearrangements within the major arc were screened by long‐range PCR to amplify a ~13.0 kb product in wild‐type mtDNA (primers: NC_012920.1 chrM:129‐110, chrM:3965‐3984). The level of deleted mtDNA in individual muscle fibers isolated by laser microcapture was determined by quantitative real‐time PCR using the ABI PRISM Step One real‐time PCR System (Life Technologies, Paisley, UK) to confirm the presence of clonally expanded mtDNA deletions.^5^

### Whole exome sequencing and bioinformatic analyses

Whole exome sequencing was performed using Illumina paired‐end precapture libraries constructed according to manufacturer's protocol. Sequence data were aligned, variants were called by GATK, and custom Perl scripts were used to annotate variants as previously described.^6^ Multiple metrics for prediction of potential functional consequences of variants were applied: CADD,^7^ Genomic Evolutionary Rate Profiling (GERP),^8^ and PhyloP. The Exome Aggregation Consortium (ExAC) Cohort was used as reference population data (http://exac.broadinstitute.org, accessed January, 2016). All genetic alleles studied were annotated in reference to *POLG2* NM_007215.

### Western blotting

Fibroblasts were grown in DMEM High Glucose with L‐Glutamine and Sodium pyruvate (Hyclone, ThermoFisherScientific) with 15% FBS. Crude mitochondrial fraction was prepared from 1 × 10^7^ cells following Graham & Rickwood method.^9^ Mitochondrial fractions were lysed in RIPA buffer containing 50 mm Tris, pH 8.0, 150 mm NaCl, 0.5% sodium deoxycholate, 1% Triton X‐100, 0.1% SDS, 5 mg/mL leupeptin, 2 mg/mL aprotinin, and 0.5 mmol/L PMSF. Thirty milligram of mitochondrial lysates were resolved on SDS‐polyacrylamide gel electrophoresis and transferred to a 0.45‐mm PVDF membrane (Millipore). Western blotting was performed as previously described.^4^

### Reverse Transcription Quantitative PCR

RNA extraction was performed using TRIzol and purified using PureLink RNA minikit (Life Technologies). First‐strand cDNA synthesis was done using SuperScript III First‐Strand Synthesis System for RT‐PCR (Life Technologies) and oligodT. Quantitative RT‐PCR experiments were performed using StepOne Plus RT‐PCR system (Applied Biosystems). *POLG2* oligonucleotides: forward 5′AAACCCTGTGGAACCTAGGAG3′ and reverse 5′AGCATGCCTCGGTCTAGGTC3′. Each sample was run in triplicate and was normalized to GAPDH.

### Membrane potential

Fibroblasts were grown on glass chamber slides and treated with 30 ng/mL rotenone for 4 h at 37°C, or the same volume of solvent. 5,5′,6,6′‐tetraethylbenzimidazolyl‐carbocyanine iodide (JC‐1, Life Sciences) of 5 *μ*g/mL was added, and the cells were incubated for 30 min at 37°C. Membrane potential was determined by the ratio of red/green fluorescence in eight random 400× microscopic fields and expressed as mean ± standard deviation (SD).

Consent for the study was obtained from all subjects and a local ethics review board approved the study.

## Results

### Clinical presentation

**Figure 1:**
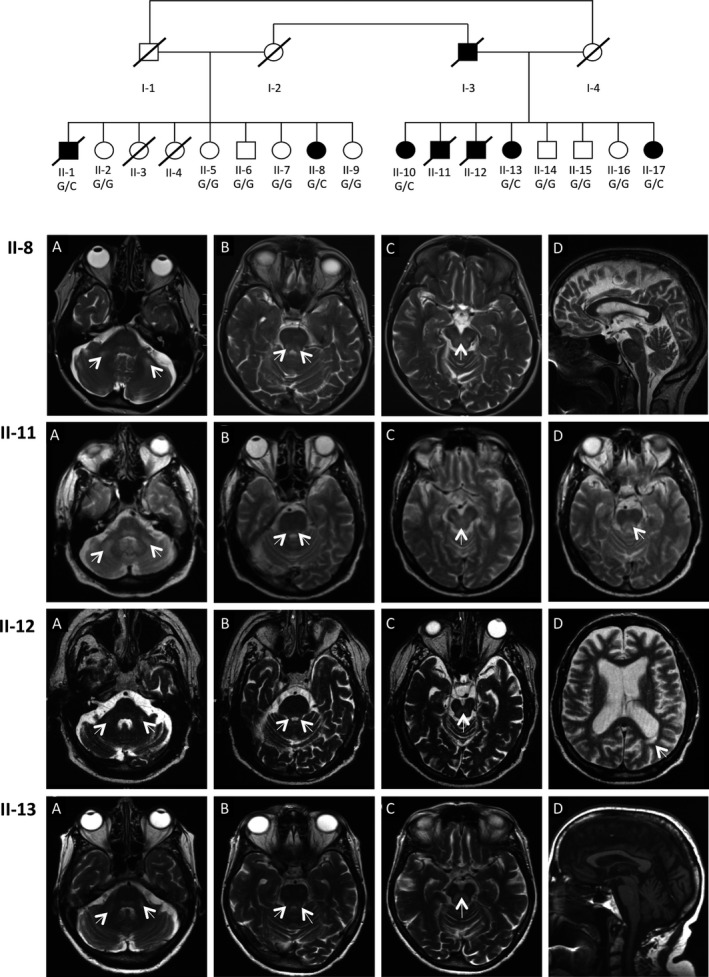
Pedigree and MRIs for family segregating *POLG2* c.970‐1G>C variant. Affected individuals are represented as black symbols, whereas unaffected are open symbols. The genotype for the *POLG2* c.970‐1G>C variant is indicated for individuals who were genotyped. Brain MRIs reveal abnormalities of the cerebellum and brain stem and white matter lesions. Axial T2‐weighted images revealed bilateral hyperintense lesions of (A) middle cerebellar peduncles, (B) upper cerebellar peduncles, and (C) periaqueductal grey matter in patients II‐8, II‐11, II‐12, and II‐13. Also shown are the following: II‐8 (D) Sagittal T2 image demonstrating mild supra and infratentorial atrophylenticular nuclei; II‐11 (D) Axial T2 image showing hypersignal of medial longitudinal fasciculus; II‐12 (D) Axial T2 image showing important hemispheric atrophy and periventricular white matter lesion; and II‐13 (D) Sagittal T1 image demonstrating moderate pontocerebellar and supratentorial atrophy.

Seven native Belgian individuals from two sibships belonging to the same extended family presented with a neurological disorder involving cerebellar and sensory ataxia with tremor (Fig. 1). Initial signs of disease rose to clinical attention between 50 and 77 years of age and in most cases resulted in loss of life in less than 15 years from onset (Table 1). Peripheral neuropathy developed in all individuals either concomitant with the cerebellar syndrome or manifesting up to 6 years postonset of disease. Additional features of disease in some individuals included epilepsy, cognitive decline, ophthalmoplegia, hearing loss, and parkinsonism. An overview of clinical and paraclinical signs is presented in Table 1 and is summarized as follows.

**Table 1:** An overview of clinical characteristics

	Subject ID						
	(II‐1)	(II‐8)	(II‐10)	(II‐11)	(II‐12)	(II‐13)	(II‐17)
POLG2 c.970‐1G>C	+	+	+	NA	NA	+	+
Gender	M	F	F	M	M	F	F
Age at onset	53	51	77	61	60	61	56
Age at death	68	Alive 69	Alive 83	69	68	77	Alive68
Cerebellar syndrome, age at onset	50	52	77	61	60	61	56
Postural and action tremor	+	+	77	61	63	63	56
Head tremor	60	55	77	−	>66	65	58
Cerebellar ataxia	+	+	77	61	60	61	58
Dysmetria			−	67	63	71	58
Peripheral nervous system, age at onset	50	52	80	67	60	61	57
Sensory loss	+	+	80	67	63	67	57
Areflexia, deep tendon	+	+	−	67	63	67	57
Sensory ataxia	+	+	−	67	63	61	57
Neuropathic pain	−	−	−	−	60	−	−
Parkinsonism, age at onset	−	−	77	−	−	65	57
Bradykinesia	−	−	77	−	−	65	57
Rest tremor	−	−	77	−	−	−	59
Rigidity	−	−	77	−	−	74	69
Postural Instability	−	−	77	−	−	67	57
Foot dystonia	−	−	77	−	−	71	59
Epileptic seizures	+	+	−	69	63	−	59
Myoclonus	−	−	−	69	63	−	−
Cognitive impairment	+	−	−	68	>66	61	57
Ophthalmoplegia	−	−	−	−	−	61	59
Ptosis	−	−	−	−	−	−	58
Hearing loss	−	−	−	−	64	67	−
Nerve conduction studies							
Sensory axonal neuropathy	+	+	−	+	+	+	+
Motor axonal neuropathy	+	−	−	+	−	−	−
MRI hypersignals of brain stem, cerebellum, hemispheric white matter	+	+	−	+	+	+	+
Muscle biopsy: mosaic COX defect	+	+	+	NA	NA	NA	+
Multiple mtDNA deletions	+	+	+	NA	NA	NA	+

NA, Not Available.

### Cerebellar ataxia, sensory neuropathy, and action tremor

Patient II‐1, presented at age 53 with gait disturbances. He experienced both cerebellar and sensory ataxia, progressing within 6 years to a loss of ambulation. Additionally, he experienced head tremor followed closely by an arm tremor that was both intentional and resting. Electrophysiological studies, comprised of recordings of nerve conduction velocities (NCV), electromyography (EMG), and somesthesic‐evoked potentials (SEPs) indicated an axonal sensorimotor neuropathy in the lower limbs, and a pure sensory neuropathy of upper limbs. SEPs indicated a near‐absent spinal response and a very low‐amplitude cortical response. Patients II‐8, II‐11, II‐12, II‐13, and II‐17 showed similar clinical signs and courses with details shown in Table 1. Available results from electrophysiology studies for all subjects are shown in Table S1.

### Parkinsonism

One subject, II‐10, displayed a significantly different clinical presentation and clinical course compared to the rest of the kinship. Her disease presented at age 77 years, two decades later than most others in this family, when she received a diagnosis of asymmetrical Parkinson's disease based on the onset of a resting tremor of the left hand, mild left‐sided rigidity, generalized akinesia (right<left), hypomimia, head tremor, mild reduction in stride length, reduced arm swing, and mild dystonic movements of the left foot. While mild tandem ataxia was observed, notably, patient II‐10 had no sensory complaint at the onset of symptoms and NCV and brain MRI were normal. In a 5‐year follow‐up exam, only moderate reduction in pallesthesia of lower limbs was noted, whereas NCV and MRI were not repeated. This subject's parkinsonian symptoms improved with levodopa treatment.

Two additional patients displayed parkinsonism in combination with ataxia and peripheral neuropathy as described above. Patients II‐13 and II‐17 had bilateral bradykinesia, reduced postural reflexes, and facilitory paratonia without resting tremor or cogwheel rigidity at the onset of their illness. In contrast to subject II‐10, these parkinsonian symptoms did not respond to levodopa.

### Seizures and cognitive impairment

Five patients (II‐1, II‐11, II‐12, II‐13, and II‐17) developed progressive cognitive impairment of the frontal‐subcortical subtype leading to profound dementia in two of them (III, 11 and III, 17). The three males II‐1, II‐11, and II‐12 had a rapidly progressive cognitive decline followed by generalized tonic‐clonic seizures a few months before death in *status epilepticus* at the end of their seventh decade. Two females II‐8 and II‐17 had isolated episodes of generalized tonic‐clonic seizures a few years after the onset of their symptoms.

### Ophthalmologic manifestations

Two patients displayed ophthalmological signs. Patient II‐17 showed classic hallmarks of PEO with ptosis but no nystagmus, at age 59. By the age of 68, oculomotor examination revealed ophthalmoplegia with moderate limitation of amplitude and marked slowness of horizontal and vertical saccades and pursuit movements. Patient II‐13 had moderate ophthalmoparesis without ptosis at 72. It is noteworthy that the age of onset of this patient's ophthalmologic dysfunction was beyond the age of death of most affected subjects in this family.

### Brain MRI reveals cortical and cerebellar atrophy with cerebellar and brainstem hypersignals

Affected individuals displayed cerebellar and brainstem abnormalities on T2‐Weighted and T2 FLAIR images with focal hyperintense lesions of middle and upper cerebellar peduncles, posterior area of the pons and mesencephalon (medial longitudinal fasciculus), and the periaqueductal grey matter. They also exhibited various degrees of multifocal hyperintense hemispheric white matter lesions, cortical atrophy, and pontocerebellar atrophy (Fig. 1). Patient II‐8 had a normal MRI at the onset of symptoms. However, a second MRI performed 6 years later showed the appearance of T2 and FLAIR diffuse hypersignals at the level of middle cerebellar peduncles and midbrain and cerebellar cortical atrophy, similar to those observed in other affected family members.

### Patient muscle shows signatures of mitochondrial dysfunction including multiple mtDNA deletions

**Figure 2:**
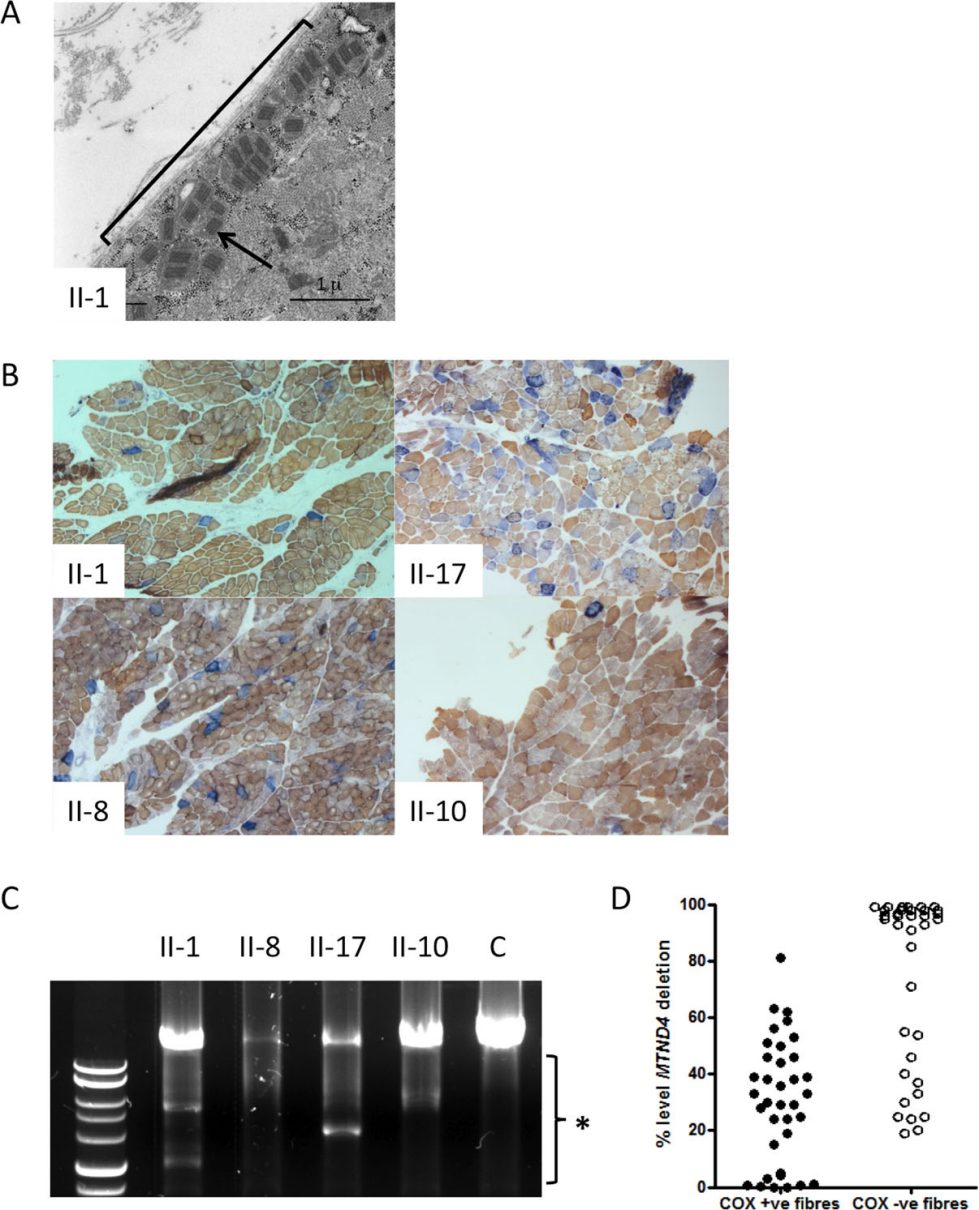
Electron microscopy, histochemistry, and mtDNA analyses in patient muscle show signs of mitochondrial dysfunction. (A) Electron microscopy of Patient II‐1 skeletal muscle reveals subsarcolemmal accumulation of abnormal mitochondria with paracrystalline inclusions. The general region of subsarcolemnal accumulation is indicated with a black bracket and a black arrow points to one of the paracrystalline inclusions. (B) Sequential COX succinate dehydrogenase (SDH) histochemistry demonstrates a mosaic distribution of COX‐deficient muscle fibers (blue) among fibers exhibiting normal COX activity (brown). Illustrated are the images for Patients II‐1, II‐17, II‐8, and II‐10. (C) Long‐range PCR across the major mtDNA shows evidence of variable mtDNA deletions in muscle from patients II‐1, II‐17, II‐8, and II‐10. The black bracket indicates the region of the gel where deletion fragments segregate. (D) Quantitative, single‐fiber real‐time PCR reveals the majority—but not all—of COX‐deficient fibers contain high levels of a clonally expanded mtDNA deletion involving the *MTND4* gene (36 COX‐positive and 36 COX‐deficient fibers laser captured from the biopsy material of patients II‐1, II‐8, and II‐17).

Skeletal muscle biopsies from four affected individuals were examined for evidence of mitochondrial dysfunction. Electron microscopy ultrastructural studies revealed several anomalies including subsarcolemmal accumulation of abnormal mitochondria with paracrystalline inclusions (Fig. 2A). Histopathological studies exhibited evidence of subsarcolemmal mitochondrial accumulation as ragged‐blue fibers following SDH enzyme histochemistry, whereas sequential COX‐SDH histochemistry revealed a mosaic pattern of COX deficiency, with preserved SDH activity, in all four patients (Fig. 2B). Interestingly, there was variability in severity of the associated COX defect with 25% COX‐deficient fibers and 5% ragged‐red fibers observed in patient II‐8, whereas patient II‐10 showed milder changes with only 2% COX‐deficient fibers and 1% ragged‐red fibers (Fig. 2B), correlating with disease severity.

Molecular studies revealed that muscle from all four patients studied showed evidence of multiple mtDNA deletions (Fig. 2C). Single muscle fibers were investigated to characterize the presence of clonally expanded mtDNA deletions, demonstrating that the majority of COX‐deficient fibers revealed very high levels (>80% mutated mtDNA) of clonally expanded mtDNA deletion, whereas all COX‐positive reacting fibers apart from one had levels of mtDNA deletion below this level (Fig. 2D).

### Genetic studies reveal *POLG2* c.970‐1G>C heterozygous variant segregates with disease

Whole‐exome sequencing identified a heterozygous splice variant, *POLG2* c.970‐1G>C, in affected individual II‐17. Variants in *POLG2* are known to cause multiple mtDNA deletions which was one of the molecular phenotypes observed in muscle biopsy from our patients. The *POLG2* c.970‐1 G>C variant has not been previously reported in the *POLG2* disease literature and is not present in ExAC database. Sequencing of family members confirmed *POLG2* c.970‐1G>C segregated with disease in this pedigree (Fig. 1).

### POLG2 displays reduced RNA and protein levels in patient fibroblasts

**Figure 3:**
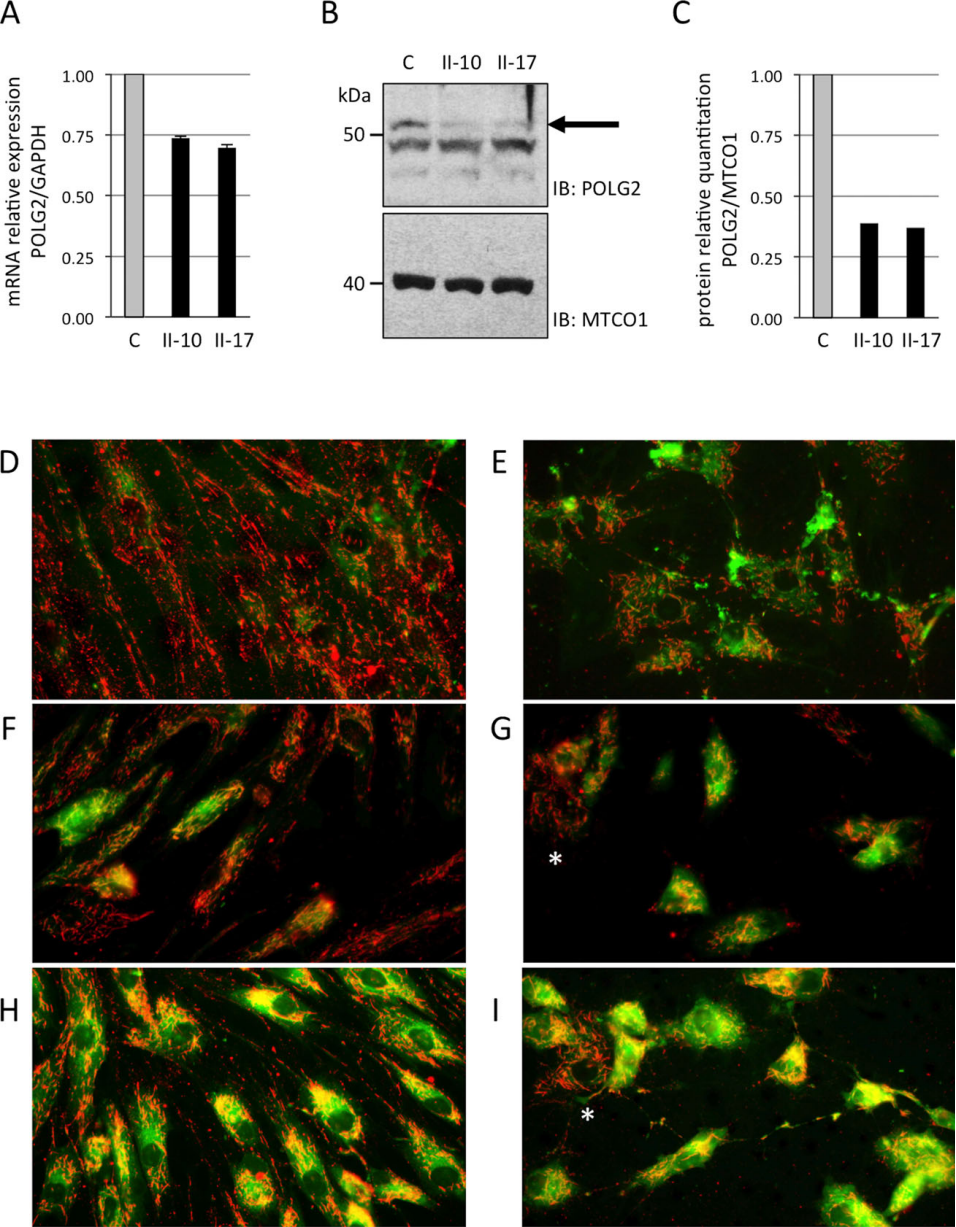
POLG2 mRNA and protein levels and mitochondrial membrane potential are decreased in patient fibroblasts. The level of POLG2 RNA and protein was measured in control (C) and patient cells (II‐10 and II‐17). (A) Quantitative real‐time PCR experiments were performed for human GAPDH and POLG2 and expressed as a relative quantitation of POLG2/GAPDH. (B) Mitochondrial lysates were analyzed by Western blotting using a rabbit polyclonal antisera raised against the recombinant human POLG2 protein. POLG2 is represented by the top band visible migrating at 55 kilodaltons (kDa), while two additional lower bands represent nonspecific binding. The black arrow points to the POLG2 band to help distinguish it from the nonspecific bands. (C) Relative quantitation of protein was measured by comparing the intensity of POLG2 and MTCO1 bands in controls and patients. D‐I. JC‐1 fluorescence studies of patient fibroblasts show reduced mitochondrial membrane potential. Imaging visualized mitochondria with inactive (green) or active (red) membrane potential. Membrane potential was assessed in untreated (D, F, H) and rotenone‐treated (E, G, I) fibroblast cells. Control fibroblasts are shown in D, E; Patient II‐1 fibroblast in F, G; Patient II‐17 fibroblast in H, I. A mosaic staining pattern of individual cells is present in patient fibroblasts, with some cells displaying predominantly red staining indicative of active mitochondria in these cells (these cells are marked with a white asterisk).

The pathogenic potential of the *POLG2* variant was tested through measuring the levels of POLG2 RNA and protein in patient cells. The *POLG2* c.970‐1G>C variant is located in the intron 4 splice acceptor site and predicted to cause skipping of exon 5. Improper splicing of a transcript may trigger nonsense‐mediated decay or nonsense‐associated splicing and result in reduced levels of mRNA and protein. Reduced amounts of *POLG2* mRNA were detected in both patient fibroblast cell lines tested (Fig. 3A). POLG2 protein levels in patient cells were 40% lower than levels in healthy control cells (Fig. 3B and C). To our knowledge this is the first example of a *POLG2* variant causing reduction in protein levels. This drastic reduction in POLG2 protein is likely the cause of the mtDNA deletions detected (Fig. 2C and D) and the pathological symptoms.

### Patient fibroblasts show deficiencies in mitochondrial membrane potential

Patient fibroblasts demonstrate a substantial reduction in mitochondrial membrane potential (Fig. 3D–I). Membrane potential was 2.18 ± 0.44 in controls (mean ± SD), whereas patients showed a substantial reduction to 1.45 ± 0.34 in II‐1, 1.71 ± 0.34 in II‐10, and 1.61 ± 0.31 in II‐17. Notably, these results reveal a reduction in mitochondrial membrane potential concordant with severity of patient clinical presentation. Inhibition of complex I activity depresses membrane potential and causes intracellular movement of the mitochondria toward the nucleus, thus further exposing bioenergetics defects. Upon addition of rotenone, a complex I inhibitor, mitochondrial membrane potential shifted down in controls and showed even greater deficits in patients’ cells. Notably, JC‐1 staining showed variability in the extent of green to red across individual cells, roughly correlating with severity of disease (Fig. 3D–I). This mosaic pattern of staining across cells has been previously observed in primary mitochondrial disease patients with combined OXPHOS deficiency due to an underlying molecular etiology that results in a variable mutational load in mtDNA across patient cells.^10^ To our knowledge this is the first demonstration of this staining pattern in cells from patients with a *POLG2* pathogenic variant as well as the first documentation of this pattern in any patient with multiple mtDNA deletions.

### Variable expressivity and the possibility of reduced penetrance

The four founders in this pedigree were not available for participation in this study as all were deceased. The mother of the first sibship (I‐2) and the father of the second sibship (I‐3) were siblings (Fig. 1). Likewise, the father of the first sibship (I‐1) and mother of the second sibship (I‐4) were siblings. Of these four individuals only one, I‐3, was noted by surviving family members as having a neurological disorder. This individual received a diagnosis of Parkinson's disease at the age of 70 and died at the age of 79. This makes I‐3 the likeliest carrier of the *POLG2* c.970‐1G>C variant among the four founders, which obligates I‐2 as a carrier as well. Individual I‐2 reportedly died in ‘older age’ without signs of neurological disease, raising the possibility of incomplete penetrance of this variant. However, all members of the second generation of the pedigree who were available for genotyping have genotypes that correspond to full penetrance of the variant, this included eight individuals who were asymptomatic and did not possess the variant. Additionally, the oldest age of disease onset in this family is 77 and at the most recent clinic visit this subject II‐10, at age 82, was the oldest symptomatic person in this study and manifested the mildest form of disease with parkinsonism, cerebellar ataxia, and sensory loss. This suggests that variable expressivity may present with this variant, as is not uncommon with *POLG* disease‐causing variants. Low levels of inherited heteroplasmic mutations of mtDNA appear to accelerate the accumulation of somatic mutation in mtDNA^11^ and this may influence variable ages of onset of clinical presentation across individuals carrying the same mutation.

## Discussion

We describe a neurological syndrome comprised of cerebellar ataxia, axonal sensory ataxic neuropathy, and tremor in combination with epileptic seizures, cognitive decline, parkinsonism, and ophthalmoplegia. Brain imaging studies indicated cerebellar and brainstem atrophy. Genetic studies identified a heterozygous *POLG2* splice acceptor variant, c.970‐1G>C, segregating with disease that caused diminished levels of POLG2 mRNA and protein in patient tissue. To our knowledge this is the first example of a *POLG2* variant causing reduction in protein levels. The clinical signs in this family are distinct from previously reported cases of POLG2 deficiency and the syndrome described here extends the *POLG2*‐related phenotypic spectrum.

Recent microscopy reveals that POLG2 expression and subcellular localization is critical for mtDNA replication.^12^ Autosomal dominant nonsynonymous point mutations in POLG2 have previously been shown to cause late‐onset PEO with mtDNA deletions.^2^, ^13^ While total reduction in *POLG2* is embryonic lethal, 14 a reduction in POLG2 by siRNA causes a 50% reduction in mtDNA in only 6 days.^15^ Mutations that affect splicing and expression in POLG2 have previously been described by Walter et al. 2010.^3^ In that patient, a heterozygous 24 bp in‐frame insertion was identified that caused skipping of exon 7 and degradation of the POLG2 message affecting overall POLG2 expression. This reduction in *POLG2* expression was associated with late‐onset ptosis and myopathy. Thus, the c.970‐1G>C splice acceptor variant in *POLG2* we identified that causes reduction in POLG2 expression is consistent with previous *POLG2* mutations causing mtDNA deletions and late‐onset mitochondrial disease.

Like *POLG2* mutations, other genetic causes of primary multiple mtDNA deletion syndromes most often present as adPEO with myopathy, but also manifest a broad range of age of onset with variable phenotypes and expressivity. The neurological syndrome described here shares some similarities in clinical presentation with a *POLG*‐related syndrome, sensory ataxic neuropathy, dysarthria, and ophthalmoparesis (SANDO).^16^ SANDO may be distinguished from this disorder by age of onset as it typically starts in childhood or early adulthood and by the inclusion of dysarthria in the SANDO clinical presentation. Likewise, variants in another member of the mtDNA replication complex, *C10orf2*, can lead to an ataxia‐neuropathy syndrome that includes dementia.^17^ Parkinsonism is a clinical feature of the neurological syndrome described here that is not commonly reported in conjunction with multiple mtDNA deletions, however, variants in *POLG,* 18, 19 *C10orf2,* 20 *OPA1,* 21 and *MPV17* 22 have been associated with neurological syndromes that include parkinsonism as a component.

A thorough consideration of diseases included in the differential diagnosis is particularly relevant given the demonstrated variable expressivity of this variant and others leading to similar phenotypes. Among additional neurological syndromes that overlap with this disorder, but do not have a primary mitochondrial etiology, a special attention should be paid to Fragile X‐associated tremor/ataxia syndrome (FXTAS), as it includes a similar age of onset with progressive intention tremor, parkinsonism, cognitive decline, and generalized cortical atrophy and hypersignals in the middle cerebellar peduncles on MRI.^23^ Taking into consideration these similarities, we suggest considering POLG2 deficiency in the differential diagnosis for cases of ataxia and sensory neuropathy in adults, especially when it is accompanied by tremor or parkinsonism with white matter disease.

The mtDNA deletions identified in the patients’ tissues in this study result from faulty replication of the mitochondrial genome due to the germline transmission of a genetic variant, *POLG2* c.970‐1G>C, that likely results in reduced efficacy of the mtDNA polymerase. mtDNA deletions may also occur in the tissues of individuals without a primary mitochondrial disorder through DNA damage induced by ROS coupled with improper repair^24^ or through faulty replication.^25^ As a result, mtDNA deletions along with a corresponding mosaic COX deficiency pattern and single‐cell clonal expansion of mtDNA deletions have also been observed in aged tissues of healthy individuals as well as in tissues from patients with neurodegeneration that is not due to a primary mitochondrial disorder. A relatively high level of deleted mtDNA has been observed in substantia nigra neurons from both aged controls and individuals with Parkinson's disease (67% of COX‐deficient fibers).^26^ Some extent of mutant mtDNA in a cell can seemingly be tolerated without an apparent functional consequence. This has been studied through multiple modes and end points and generally a mutational load of 60–90% of mtDNA molecules within a single cell must be mutated before functional consequences are appreciated.^27^ In this study, approximately 25% of patient muscle fibers tested were COX deficient and of these individual fibers typically showed >80% mutant mtDNA molecules. The germline transmission of a pathogenic variant in a gene essential for proper maintenance of the mitochondrial genome leads to the accumulation of somatic mtDNA deletions which may result in the same clinical end points, such as Parkinson's disease, as manifest in individuals who do not possess a germline variant but rather have accumulated somatic mtDNA deletions for other reasons (DNA damage induced by ROS or faulty replication). These studies converge around the fundamental concept of accumulation of somatic mtDNA deletions playing a crucial role in the neurological decline observed in brain aging and in Parkinson's disease, regardless of etiology.

## Author Contributions

Conception and design of the study: LVM and PEB. Acquisition and analyses of data: PEB, AB, VA, BDP, RVC, RWT, ELB, LH, KC, GS, MG, AR, GD, PL, PH, JJM, JB, MMH, and FGD. Drafting the manuscript or figures: PEB, LVM, WCC, JP, RWT, BDP, AB, and VA.

## Conflict of Interest

The authors declare no competing interests.

## Supporting information


**Table S1.** Electrophysiological studies: conduction studies on sensory and motor nerves.
