# Supplementary material for: POLG2 deficiency causes adult‐onset syndromic sensory neuropathy, ataxia and parkinsonism
Source: Ann Clin Transl Neurol. 2016 Nov 16;4(1):4–14. doi: 10.1002/acn3.361 (PMC5221457; doi:10.1002/acn3.361)
Supplement: Supplementary file 1 — Table S1. Electrophysiological studies: conduction studies on sensory and motor nerves. [file ACN3-4-4-s001.doc]

**Supplemental Table 1**. Electrophysiological studies: conduction studies on sensory and motor nerves.

| Nerve | Normal value | Patient  II, 1 | Patient  II, 8 | Patient  II, 10 | Patient  II, 11 | Patient  II, 12 | Patient  II, 13 | Patient  II, 17 |
| --- | --- | --- | --- | --- | --- | --- | --- | --- |
| Age |  | 58 | 62 | 78 | 69 | 64 | 67 | 60 |
| Sensory |  |  |  |  |  |  |  |  |
| **Right median** |  |  |  |  |  |  |  |  |
| Amp (μV) | > 20 | **ND** | **ND** | NA | NA | **ND** | NA | NA |
| CV (m/s) | > 50 |  |  | NA | NA |  | NA | NA |
| **Left median** |  |  |  |  |  |  |  |  |
| Amp (μV) | >20 | **ND** | **ND** | NA | NA | **ND** | NA | NA |
| CV (m/s) | > 50 |  |  | NA | NA |  | NA | NA |
| **Right radial** |  |  |  |  |  |  |  |  |
| Amp (μV) | > 15 | **ND** | **2.7** | NA | NA | NA | NA | NA |
| CV (m/s) | > 50 |  | NA | NA | NA | NA | NA | NA |
| **Left radial** |  |  |  |  |  |  |  |  |
| Amp (µV) | > 15 | **ND** | **1.4** | NA | NA | NA | NA | NA |
| CV (m/sec) | > 50 |  | NA | NA | NA | NA | NA | NA |
| **Right sural** |  |  |  |  |  |  |  |  |
| Amp (μV) | > 6.0 | **ND** | **ND** | 8.1 | **ND** | **ND** | **ND** | **ND** |
| CV (m/s) | >40 |  |  | 41 |  |  |  |  |
| **Left sural** |  |  |  |  |  |  |  |  |
| Amp (μV) | > 6.0 | **ND** | **ND** | 5.1 | **ND** | **ND** | **ND** | **ND** |
| CV (m/s) | > 40 |  |  | 40 |  |  |  |  |
| Motor |  |  |  |  |  |  |  |  |
| **Right median** |  |  |  |  |  |  |  |  |
| d-Amp (mV) | > 5.0 | 6.4 | 10.6 | NA | NA | 18.6 | NA | NA |
| CV (m/s) | > 50 | 55 | 55 | NA | NA | 56 | NA | NA |
| **Left median** |  |  |  |  |  |  |  |  |
| d-Amp (mV) | > 5.0 | 6.4 | NA | NA | NA | 17.5 | NA | NA |
| CV (m/s) | > 50 | **46.9** | NA | NA | NA | 47 | NA | NA |
| **Right peroneal** |  |  |  |  |  |  |  |  |
| d-Amp (mV) | > 2.0 | **0.216** | 5.2 | 3.9 | **1.7** | **1.5** | 2.9 | 3.0 |
| CV (m/s) | > 40 | **33.9** | 45 | 49 | 42 | 46 | 50 | 46 |
| F waves (ms) |  | **ND** | 44 | NA | NA | NA | NA | 45.9 |
| **Left peroneal** |  |  |  |  |  |  |  |  |
| d-Amp (mV) | > 2.0 | **0.206** | 7.5 | 4.6 | 3.7 | 3.8 | 2.8 | 2.9 |
| CV (m/s) | > 40 | **38** | 45 | 45 | 41 | 46 | 56 | 44 |
| F waves (ms) |  | **ND** | 45.3 | NA | NA | NA | NA | 44.7 |
| **Right tibial** |  |  |  |  |  |  |  |  |
| d-Amp (mV) | > 4.0 | **0.301** | 6.9 | 12.5 | **0.9** | **2.9** | 8.6 | 10.6 |
| CV (m/s) | > 39 | 40.2 | 44 | 43 | 41 | 48 | 49 | 43 |
| F waves (ms) |  | **ND** | 48.3 | NA | NA | NA | NA | NA |
| **Left tibial** |  |  |  |  |  |  |  |  |
| d-Amp (mV) | > 4.0 | **0.461** | 4.9 | 8.1 | **3.7** | **2.3** | 8.2 | 7.9 |
| CV (m/s) | > 39 | **37** | 46 | 49 | 41 | 46 | 49 | 44 |
| F waves (ms) |  | **ND** | 44.9 | NA | NA | NA | NA | NA |

Amp = amplitude. μV = microvolt. CV = conduction velocity. mV = millivolt. d-Amp = distal amplitude. ms = millisecond. NA = non available. ND = non detectable. **Bold** values are abnormal.
